# A Singular Case of Congenital Misplacements in a Dog

**Published:** 1884-01

**Authors:** 


### CASE DEPARTMENT.

### I.—A SINGULAR CASE OF CONGENITAL MISPLACEMENTS IN A DOG.

*History.*—The patient was a thoroughbred cocker-spaniel about three years old. It was brought to me for examination by two of my colleagues, one of whom had charge of it. This gentleman looked upon the condition as being primarily that of "sarcocele," with the secondary development of "hydrocele." So far as be could gain any information, the condition had been noticed by the owner some three months. The cause, which necessitated calling in professional attendance, was difficulty in defecation, united with much pain.

*Diagnosis.*—A subcutaneous movable tumor on the right side of the raphé, partially in the perineal region, but mostly to the right of it, probably of a cancerous nature.

*Status præsens.*—Body well nourished; general health good; right testicle not to be felt. The tumor was oval, about two and one-half inches long by one and a half broad; on palpation one felt a soft yielding mass somewhat like dough in consistency, occupying the superior part of the tumefaction; aside from this, an elongated, ovoid object could be felt which was movable in all directions. This object was looked upon by my colleagues as the missing tubicle. At the time of my examination nothing indicating the presence of fluid within the sac could be felt, but that the gentlemen were not mistaken in their diagnosis of its presence, at intervals, will be shown shortly.

*Operation.*—"The operation was successful, but the patient died." Some would probably be of the opinion, more especially veterinarians, that it is a poor policy to chronicle one's errors in diagnosis, or surgery, but it is my opinion that the careful study of them is of fully as much benefit to myself, and others as reporting our success.

A complete anesthesis was produced by ether. On a second and very careful examination we saw no reason for changing our conclusions. Section of the skin being made we were enabled to examine more critically the contents of the tumefaction. *No fluid escaped from the sac on opening it.* There were no adhesions between the internal walls of the sac and its contents. The dough-like mass was found to consist of adipose tissue; the hard object was about the size of a man's testicle, somewhat flattened, with a sort of line of demarcation, or indentation, indicating a median septum of tissue, more dense than the remainder of the body, which had a somewhat spongy feeling, the surface of this object was quite rough, slightly nodulated; on its posterior inferior surface one could feel a cystoid appendage which did not contain any perceptible amount of fluid. One could feel quite a number of, apparently, ligamentous connections between this object and the body. On further examination of the adipose tissue we could follow it into a canal which extended into the abdominal cavity, along the course of the rectum; the canal would easily admit the two first fingers of a man's hand for their entire length; the fatty tissue was determined to be an omental hernia. On enucleation of the rest of the contents of the sack we found the connections to consist of two white cords; a flat ligamentous band and several blood vessels. The cystoid attachment to the tumor was conjectured to be the bladder, the two white, round cords, to be the ureters, and on catheterization the ligamentous connection proved to be the urethra, showing our conjecture to be correct as to be the presence of the bladder.

The patient having suffered a great deal during the past two weeks during defecation, due, in all probability, to a duplicature of the rectum getting into the previously mentioned canal, and its future existence promising to be connected with still more pain, it was considered justifiable to continue the anæsthetic until death was produced.

Further examination revealed the discontinuance of the penis at the angle of the ischium where it was lost in its muscular adhesions, no part of it extending over the arch into the abdominal cavity. The urethra and two blood vessels extended in a direct line, posteriorly, to the tumor, which was the hypertrophied prostrate gland. The two other vessels connected with the latter and the ureters came out through the previously mentioned canal. The missing testicle was found in the external injuinal region, atrophied and buried in adipose tissue. On opening the abdominal cavity the right kidney was found to be floating with connections six inches in length. An adipose surroundings were wanting. Other parts normal. The presence of the bladder in the sack will explain the presence of water, at times, to my colleagues, but no one seems to have noticed any variation in the external appearance of the tumor which must certainly have taken place. The increase of the amount of the omentum within the sac, and the continued enlargement of the prostate will explain the gradual increase in size of the tumefaction. The long hair on the parts sufficiently explains the reason of the tumor not being observed by the owner earlier, or until the difficulty in defacation was noticed.

B.

BOSTON. MASS.

